# Primary choriocarcinoma of the colon: a case report and review of the literature

**DOI:** 10.1186/1477-7819-11-23

**Published:** 2013-01-28

**Authors:** Lun Jiang, Jing-Tao Wu, Xin Peng

**Affiliations:** 1 Department of Medical Imaging, Su Bei People’s Hospital of JiangSu Province, Medical School of Yangzhou University, No. 98 NanTong West Road, Yangzhou, Jiangsu Province, 225001, China

**Keywords:** Adenocarcinoma, Chemotherapy, Choriocarcinoma, Colon, Syncytiotrophoblastic differentiation

## Abstract

Choriocarcinoma usually arises in the uterus and gonads. Primary choriocarcinoma (PCC) in an extragenital organ is rare. When it occurs in the gastrointestinal tract, the stomach is the most common site. Only 12 cases of PCC of the colon have been reported in the world literature. Most cases were associated with adenocarcinoma. We report the case of a 36-year-old man with PCC of the colon and review the clinical characteristics of previously documented cases.

## Background

Choriocarcinoma is a highly malignant tumor of trophoblastic cells that most often arises in the female genital tract. It is considered to be pertinent to molar pregnancy, normal or ectopic pregnancy and abortion. When it occurs in male patients, the testis is the most common site. Choriocarcinoma of extragenital origin is found in the retroperitoneum or the mediastinum, or intracalvarium. Primary choriocarcinoma (PCC) originating in the colon is extremely rare and the prognosis is usually poor.

## Case presentation

**Figure 1:**
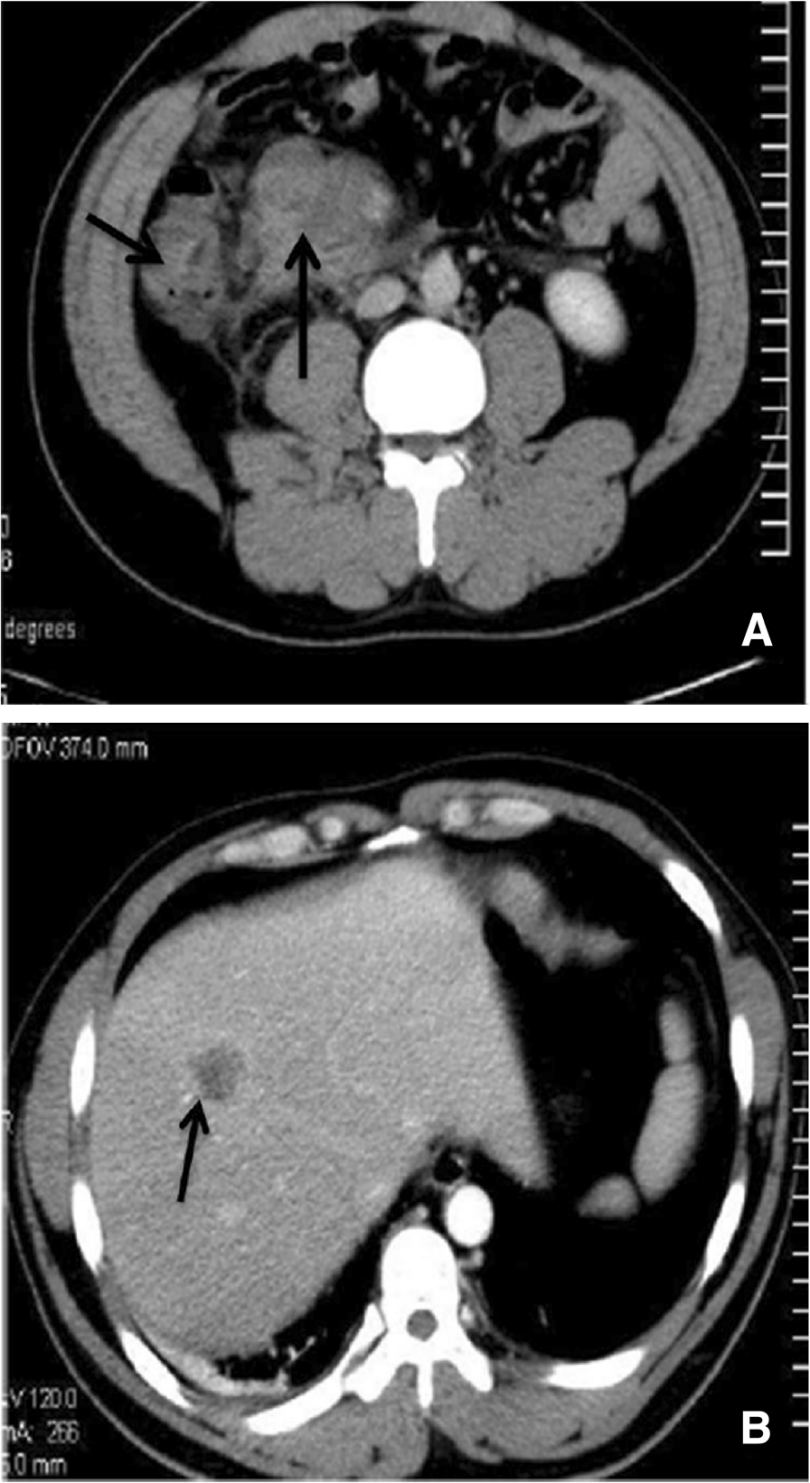
**Computed tomography. (A)** Note the thickened wall of the ascending colon (short arrow) and enlarged lymph nodes in the mesentery (long arrow). **(B)** The hepatic lesion manifested ring-shaped enhancement (arrow).

A 36-year-old male patient visited the local hospital for the chief complaint of discomfort of the upper abdomen. An appendectomy was performed under the clinical diagnosis of acute appendicitis. During the operation, a tumor was observed in the colon. The operation was aborted because of an inability to remove the tumor. Two days later, a computed tomographic (CT) scan of the abdomen revealed the mass arising from the ascending colon. The regional enlarged lymph nodes and metastases in the liver were noted (Figure 1).

**Figure 2:**
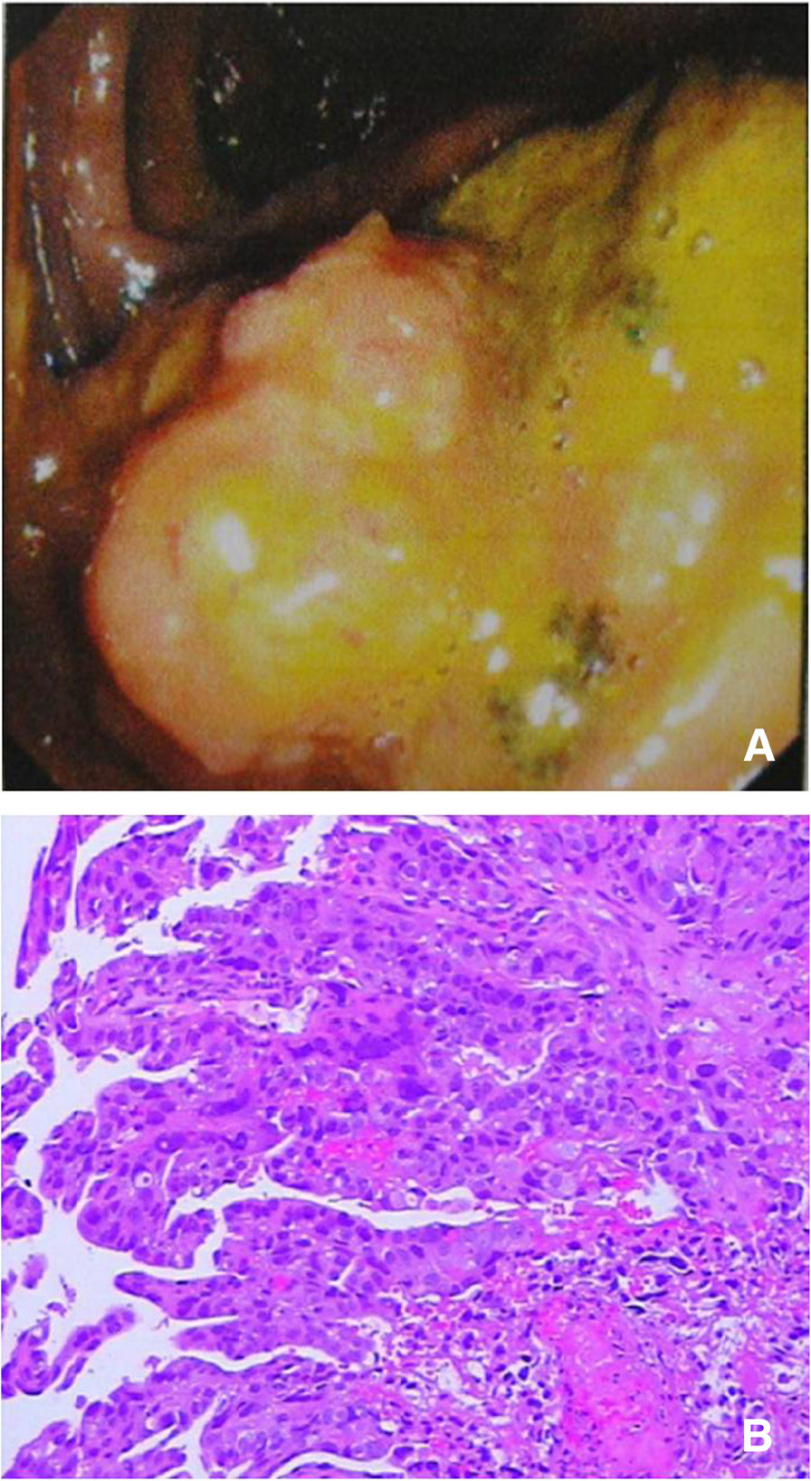
**Endoscopic biopsy (A) Colonoscopy revealed a circular yellow tumor in the ascending colon.(B)** An endoscopic biopsy revealed atypical epithelium (hematoxylin and eosin ×100).

**Figure 3:**
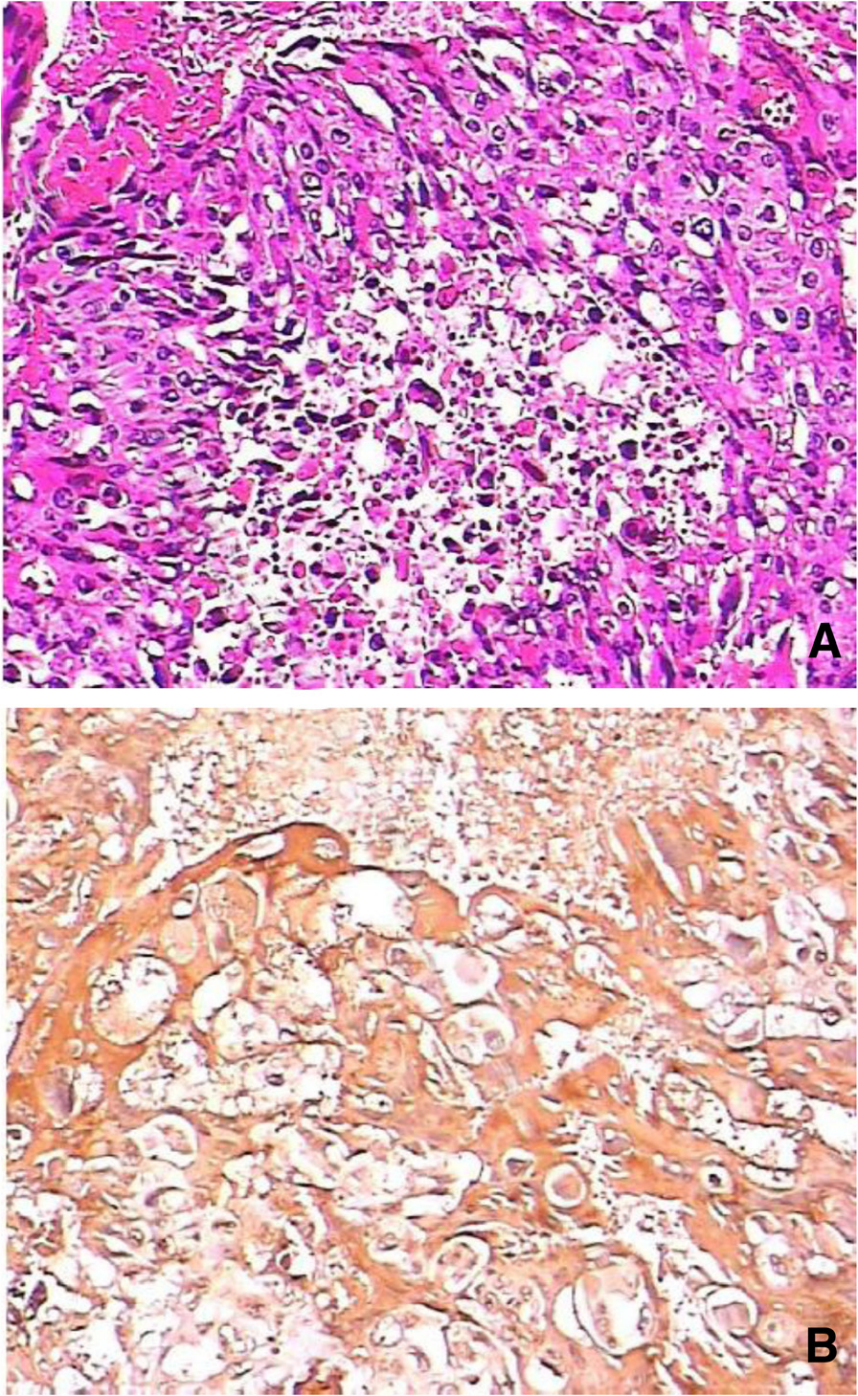
**Pathologic findings.(A)** Pathologic findings of the resected specimen revealed polynuclear giant cells. **(B)** Immunohistochemical staining was positive for human chorionic growth hormone.

The patient was transferred to our hospital for treatment. Tumor markers, including carcinoembryonic antigen, cancer antigen 19–9 (CA 19–9) and CA 125, were in the normal range. His serum beta human chorionic growth hormone (β-HCG) level was 3.38 mIU/ml. A colonoscopy revealed a yellow tumor in the colon (Figure 2) and endoscopic biopsy findings suggested a poorly differentiated adenocarcinoma. After two days, a colectomy was performed. The tumor located in the ascending colon measured 4 cm × 5 cm and penetrated the serosa and the mesocolic fat, with 12 adjacent enlarged lymph nodes. The pathologic findings showed that the tumor invaded the serosa of the intestinal wall and was composed of syncytiotrophoblastic cells, cytotrophoblast-like cells and intermediate trophoblastic cells. Necrosis and hemorrhage were also noted in the mass. Immunohistochemical staining was positive for HCG and negative for cytokeratin 7, cytokeratin 20, villin, caudal type homeobox 2, α-fetoprotein and CD30 (Figure 3). Metastasis was found in the liver and in eight of the excised lymph nodes.

Systemic chemotherapy using bleomycin, etoposide and platinum was performed. Chemotherapy engaging etoposide phosphate 100 mg, cisplatin 30 mg, bleomycin 18 mg was initiated. The β-hCG level increased from 3.38 to 10,000 mIU/ml after the first course of chemotherapy, and CT showed another mass in the liver. After three cycles of chemotherapy, his β-hCG level remained high. Because of the progression of disease, the patient and his family abandoned other alternative regimens of chemotherapy.

## Discussion

Choriocarcinoma most commonly arises in trophoblastic tissue following gestational events such as molar pregnancy, normal or ectopic pregnancy, and abortion. When the tumor occurs in men, the gonads are the most common sites. Choriocarcinoma of extragenital origin has been reported in the retroperitoneum [1], mediastinum [2], and intracalvarium (especially in the pineal gland [3]). When it occurs in the gastrointestinal tract, the stomach is the most common site [4]. PCC originating in the colon is extremely rare, with only 12 documented cases. In the literature, the mean age of presentation with PCC of the colon is 51.4 years, and the ratio of female to male is 1.6:1. Of the cases reported, 61.5% of patients presented with the tumor in the proctosigmoid; the tumor was located in the ascending colon in three patients (Table 1).

**Table 1:** Clinical characteristics of primary choriocarcinoma of colon

**Case**	**Sex and age (years)**	**Location**	**HCG (mIU/ml)**	**Metastases on admission**	**Associated with adenocarcinoma?**	**Treatment**	**Survival time**
Park and Reid 1980 [5]	F/49	S	NS	Liver lung	Y	Surgery	1 month
Nguyen 1982 [6]	M/74	S	400	None	Y	Surgery	10 weeks
Ordonez and Luna 1984 [7]	F/35	Cecum	1,612	Regional lymph nodes, liver	Y	Surgery	2 months
Kubosawa *et al*. 1984 [8]	F/50	S	230,000	None	Y	Surgery	4 months
Metz *et al*. 1985 [9]	F/42	S	154,000	Regional lymph nodes, liver, lung spleen	Y	None	
Lind and Haghighi 1986 [10]	M/42	A	610,000	Liver	N	Surgery	1 month
Tokisue *et al*. 1996 [11]	F/29	R	49,000	Lung	Y	Surgery, chemotherapy	11 months
Kiran *et al*. 2001 [12]	M/68	R	700,000	Regional lymph nodes, liver	Y	None	
Le *et al*. 2003 [13]	M/73	A	146,000	Liver, lung, brain	N	None	10 days
Verbeek *et al*. 2004 [14]	F/54	R	6,831(P)	Liver, lung	Y	Surgery, chemotherapy	8 months
Froylich *et al*. 2010 [15]	F/57	Descending colon	13,000 (P)	Lung	N	Surgery, chemotherapy	16 months
Harada *et al*. 2012 [4]	F /58	S	2,420	None	Y	Surgery, chemotherapy	More than 60 months
Present case 2012	M/38	A	3.38	Regional lymph nodes, liver	N	Surgery, chemotherapy	More than 6 months

A: ascending colon; C: cecum; F: female; M: male; NS: data not shown; P: postoperatively; R: rectum; S: sigmoid colon.

The pathogenesis of primary extragenital choriocarcinoma is controversial. Several hypotheses have been proposed, including that it develops from retained primordial germ cells that migrated abnormally during embryonic development [16]; metastasis from a latent primary lesion in the genitalia [4]; and the retrodifferentiation of pre-existing colonic carcinoma [8]. Among them, the retrodifferentiation theory from pre-existing colonic adenocarcinoma is well accepted. Given that PCC of the colon associated with adenocarcinoma accounts for 69.2% of reported cases, this theory is considered to be logical. However, concurrent adenocarcinoma was not present in our patient nor in two of the previous cases [10,13]. An explanation for choriocarcinoma unaccompanied by adenocarcinoma could be that the tumor arises directly from malignant change in the ectopic chorion or totipotent cells [13].

The preoperative diagnosis of PCC of the colon is poor. In our case, the patient was assessed as having metastatic colonic cancer. An endoscopic biopsy supported this possibility because of an insufficient specimen. However, on postoperative biopsy, he was re-diagnosed with PCC of the colon. The diagnosis of primary extragenital choriocarcinoma should firstly rule out any abnormal findings in the uterus or genitalia. The presence of cytotrophoblast and syncytiotrophoblast on biopsy, the presence of β-hCG immunoreactive cells, and elevation of serum β-hCG level confirm the diagnosis.

The majority of patients with gastric choriocarcinoma are treated surgically. Umemori *et al*. found that resection followed by adjuvant chemotherapy was the best treatment of PCC of the lung [16]. For patients with widespread metastasis, resection has proven ineffective in prolonging survival. In patients without metastasis, a combination of radical surgery and appropriate adjuvant chemotherapy contributed to their long-term survival [4]. The chemotherapy modality for primary extragenital choriocarcinoma has not been established; most treatment options are based on experience. The chemotherapy regimens for choriocarcinoma and adenocarcinoma are different. Chemotherapy for both the choriocarcinoma and the colonic adenocarcinoma is most logical according to a study by Kubosawa *et al.*[4] – their patient showed the longest survival.

The correlation between the β-hCG level and the effectiveness of treatment has not been detailed in most previous reported cases. β-hCG did not decrease to normal levels after surgery, implying that micrometastatic disease existed [17]. The reduction of β-hCG in the serum indicates response to treatment. After the resection of the primary lesion, the serum β-hCG level in our case did not decrease to normal range. Abdominal CT showed hepatic lesions when the serum β-hCG increased. We suggest the serum β-hCG level could be used as a marker when assessing the effectiveness of treatment.

## Conclusions

The prognosis of PCC of the colon is poor. In our analysis of the literature, the median survival time was less than 1 year. PCC of the colon is usually not identified until the tumor has generalized metastasis. The great majority of patients die from liver failure.

## Consent

Written informed consent was obtained from the patient for publication of this manuscript.

## Abbreviations

β-HCG: beta human chorionic growth hormone; CA: cancer antigen; CT: computed tomography; PCC: primary choriocarcinoma.

## Competing interests

The authors declare that they have no competing interests.

## Authors’ contributions

Jing-Tao Wu and Lun Jiang proposed the study. Jing-Tao Wu, Lun Jiang and Xin Peng - wrote the manuscript. Lun Jiang and Xin Peng contributed equally to this work. All authors read and approved the final manuscript.
